# Professional burnout and its correlates in Polish donor transplant coordinators

**DOI:** 10.1007/s10561-019-09787-2

**Published:** 2019-09-27

**Authors:** Marcin Bury, Hanna Rozenek, Artur Kamiński, Jarosław Czerwiński, Stanisław Wójtowicz, Jolanta Banasiewicz, Krzysztof Owczarek

**Affiliations:** 1 National Centre for Cell and Tissue Banking, Chalubinskiego 5, 02-004 Warszawa, Poland; 2 Department of Psychology and Medical Communication, Medical University of Warsaw, Zwirki i Wigury 81, 02-091 Warszawa, Poland; 3 Department of Transplantology and Central Tissue Bank, Medical University of Warsaw, Chalubinskiego 5, 02-004 Warszawa, Poland; 4 Department of Emergency Medicine, Medical University of Warsaw, Lindleya 4, 02-005 Warsaw, Poland; 5 Polish Transplant Coordinating Centre, al. Jerozolimskie 87, 02-001 Warsaw, Poland

**Keywords:** Transplant coordinators, Burnout, Psychosocial stress at work, Death anxiety, Sex differences, Relationship status

## Abstract

Job demands-resources (JD-R) model of professional burnout states that job demands predict the feeling of *exhaustion*, and lack of job resources—*disengagement* from work. This research project investigated professional burnout and it correlates, including sex, death anxiety, and relationship status in 108 Polish donor transplant coordinators involved in organ, tissue, and cell transplantations. This study employed the Polish version of the Oldenburg Burnout Inventory which follows the JD-R model, the Psychosocial Working Conditions Questionnaire—a Polish instrument based on the model of job stress proposed by Karasek—and the Polish version of the Fear of Death and Dying Questionnaire. The results were suggestive of average levels of job stress and burnout in the studied population, with men being more disengaged than women. Participants who were in relationship had significantly higher levels of exhaustion than those who were single. Exhaustion was positively correlated with years of working as a transplant coordinator but not with participants’ age. Multiple negative correlations were detected between exhaustion/disengagement and different aspects of job control, social support, and well-being. Moreover, positive correlations between different components of fear of death and dying and exhaustion were detected. Our findings, linking fear of death and dying with some aspects of professional burnout in transplant coordinators, suggest that a pre-employment screening for the level of death anxiety in candidates for transplant coordinators could be useful as this job provides chronic exposure to mortality cues.

## Introduction

The most popular concept of professional burnout describes this phenomenon as a three-dimensional syndrome. Maslach and Schaufeli (2001) have conceptualized job burnout as a psychological condition characterized by (1) exhaustion, (2) cynicism and feeling of being detached from one’s job, and (3) subjective sense of ineffectiveness and reduced personal accomplishment. Job demands-resources model (JD-R) is an alternative model which states that excessive job demands and lack of job resources lead to the development of professional burnout. Excessive job demands predict the feeling of *exhaustion*, and lack of job resources—*disengagement* from work (Demerouti et al. 2001). The JD-R model has been supported empirically; a research by Bakker and Demerouti (2003) described positive correlations between job demands and the exhaustion component of burnout, and job resources and professional efficacy, and a negative correlation between job resources and cynicism.

One of the vocational groups especially prone to experiencing burnout are health care professionals. Numerous research projects have investigated burnout in medical doctors (Grassi and Magnani 2000; Pompili and Innamorati 2010; Sanchez-Reilly et al. 2013; Shanafelt et al. 2002), nurses (Jesse et al. 2015; Leiter and Laschinger 2006; Sabo 2008), military medical personnel (Adler et al. 2017), emergency medical responders (Ballesteros Pena et al. 2008), and employees of cell and tissue banks (Kamiński et al. 2018). Some studies have also looked into professional burnout in a variety of transplant coordinators populations (Gruener 2006; Harmanci Seren et al. 2013; Kim 2013).

Research projects investigating burnout in non-medical professions reported varied degrees of sex differences in burnout level across different occupational groups (Adekola 2010; Antoniou et al 2006; Heidari 2013; Innstrand et al 2011; Norlund et al. 2010; Olanrewaju and Chineye 2013). However, we were unable to find any publications investigating sex differences in transplant coordinators’ burnout. Moreover, working as a transplant coordinator includes daily contact with death and dying and, for this reason, it may be suspected that this situation considerably influences the level of death anxiety in this vocational group. Interestingly, even though there are some studies investigating death anxiety in health care workers (Melo and Oliver 2011), we were unable to find publications dealing with this issue in the population of transplant coordinators.

This research project has been aimed at investigating professional burnout and it correlates, including sex, death anxiety, and relationship status in a cohort of Polish transplant coordinators involved in organ, tissue, and cell donation. This study employed the Polish version of the Oldenburg Burnout Inventory (OLBI) (Baka and Basińska 2016; Demerouti et al. 2001), which follows the JD-R model and measures exhaustion and disengagement as the two dimensions of burnout (Demerouti et al. 2001). Moreover, we used the Psychosocial Working Conditions Questionnaire (PWC; Widerszal-Bazyl and Cieślak 2000), a Polish instrument based on the model of job stress proposed by Karasek (1979), Karasek and Theorell (1990) and used for monitoring psychosocial stress at work. In addition, we used the Polish version (Rolinska 1994; Jastrzebski 2001) of the Fear of Death and Dying Questionnaire (FVTS, Furcht vor Tod and Sterben), a multi-dimensional German scale created by Ochsmann (1993).

## Materials and methods

Participation in this anonymous study was completely voluntary. Participants received no compensation for taking part in this research project. The study measures were filled out by participants of a conference for transplant coordinators held in Warsaw, Poland in December 2017. The sample included a large cohort of transplant coordinators involved in organ, tissue, and cell transplantations.

The research data were collected by means of the OLBI, the PWC, the FVTS, and an additional questionnaire created specifically for the purpose of collecting supplementary information from the participants.

As described in our previous publication (Kamiński and Rozenek 2018), “the Polish version of the OLBI (Baka and Basińska 2016) follows the structure of the original version (Demerouti et al. 2001) and consists of 16 items. The Inventory is comprised of 2 subscales measuring exhaustion and disengagement. Each subscale consists of 8 items, and each item is rated on a 4-point Likert scale from 1—*I agree* to 4—*I disagree*. In each subscale, the sum of total points is divided by the number of items in the scale (8) to yield a raw score. The final raw score range is from 1 to 4, with higher scores reflecting higher “exhaustion” and “disengagement”. The raw scores are then transformed into stanines. In the Polish version, the Cronbach’s α is 0.73 for the exhaustion and 0.69 for the disengagement subscale, and the test–retest reliability is 0.73 for the exhaustion and 0.67 for the disengagement subscale (Baka and Basińska 2016).”

In this study, we also used three scales of the PWP (Job Control, Social Support, and Well-Being). As described by us previously (Kamiński and Rozenek 2018), “the PWC consists of 5 theoretical, construct-based scales—Job Demands, Job Control, Social Support, Well-Being, and Desired Changes—which contain a number of empirically derived subscales. Each item is rated on a 5-point Likert scale from 1 to 5. In each scale, the sum of total points is divided by the number of items in the scale to yield raw scores (higher scores reflect higher intensity of the construct being measured), which are later transformed into stens. In the validation sample, the scales Cronbach’s α varied from 0.82 to 0.94, and Pearson r for Test–Retest reliability varied from 0.66 to 0.76. The PWC authors provide sten norms for the 8 occupational groups included in the validation sample (bank and insurance specialists, middle-range medical personnel, construction workers, shop assistants, government and self-government administration officers, computer scientists, public transport drivers, and teachers) (Widerszal-Bazyl and Cieślak 2000).”

The FVTS is a multi-dimensional German scale created by Ochsmann (1993), and in this research project we used the Polish version of this questionnaire (Rolinska 1994; Jastrzebski 2001). The FVTS consists of 6 empirical subscales: L1—Fear of Meeting with Death; L2—Fear of Mortality; L3—Fear of the End of Life; L4—Fear of Physical Destruction; L5—Fear of Life after Death; and L6—Fear of the Process of Dying. The higher the total raw score on each of the subscales, the higher the fear measured by the subscale. By adding up the scores obtained in each of the subscales, one can obtain the general index of fear of death (GI); the higher the score, the higher the general fear of death. The total score ranges from 0 to 16 for each of the subscales, and from 0 to 96 for the GI. The test–retest reliability for the Polish version of the FVTS ranged from 0.87 to 0.97 for the subscales (L1–L6), and was 0.98 for the general index (GI) (Rolinska 1994).

The statistical analysis was performed with PASW Statistics version 20. For descriptive statistics, mean (M), standard deviation (SD), and sample size (N) were obtained for each variable. Besides descriptive statistics, the statistical analysis consisted of two-sample (independent-samples) t-tests used to compare two groups, Kolmogorov–Smirnov test used to compare a sample with a reference probability distribution, and correlation analyses carried out by means of the Pearson and Tau-b Kendall correlations. The categorization of the strength of correlation coefficient was based on Dancey and Reidy (2004). For all calculations, the level of significance was set at 0.05.

## Results

The study sample consisted of 108 participants, i.e., transplant coordinators involved in organ, tissue, and cell donation. This group included 36 men (33%) and 73 women (67%), with mean age of 45.32 years (SD = 8.41). Their average time of working as a donor transplant coordinator was 7.73 years (SD = 5.26). Sixty-seven percent of participants were in a relationship while 33% declared to be single.

The results of the PWC are presented in Table 1. Majority of the scales and subscales scores fall in the Average range (stens 5–7). The highest-scored are the Job Control—Behavioral and Well-Being—Physical subscales (5.52; Average range), while the lowest-scored is the Social Support—Co-Workers subscale (4.75; Below Average range).

**Table 1:** Results for PWC scales (bolded) and subscales (N—sample size; M—mean (stens); SD (s)—standard deviation of sten results)

	N	M (r)	M (s)	SD (s)
**Job control**	107	3.17	5.21	1.87
Job control—behavioral	107	2.71	5.52	1.99
Job control—cognitive	108	3.74	4.92	1.78
**Social support**	107	2.92	4.85	2.11
Social support—supervisors	107	2.83	5.02	2.12
Social support—co-workers	107	2.94	4.75	2.12
**Well-being**	106	3.69	5.19	1.68
Well-being—physical	106	3.88	5.52	1.81
Well-being—psychological	106	3.51	5.05	1.85

The OLBI yielded the following results: a mean stanine of 4.36 (SD = 1.78) (mean raw score of 2.12, SD = 0.49) for the Disengagement scale, and a mean stanine of 4.92 (SD = 1.53) (mean raw score of 2.28, SD = 0.41) for the Exhaustion scale. Both OLBI results fall in the Average range.

The correlations between the Exhaustion or Disengagement and the PWC scales and subscales are presented in Table 2. All of the detected correlations are negative, and majority of them fall in the Weak range. There are also moderate correlations between Exhaustion and Well-Being (r =  − 0.583, *p* = 0.0001), and Disengagement and Well-Being—Psychological (r =   − 424, *p* =  0.0001). The only strong correlation was detected between Exhaustion and Well-Being—Psychological (r =  − 0.665, * p*  = 0.0001).

**Table 2:** Pearson correlations of OLBI scales and PWC scales and subscales (r—correlation coefficient; p—p-value; N—sample size)

	Exhaustion			Disengagement		
	r	*p*	N	r	*p*	N
**Job control**	− .231	0.02	102	− .336	0.001	102
Job control—behavioral	− .192	0.053	102	− .292	0.003	102
Job control—cognitive	− .263	0.007	103	.312	0.001	103
**Social support**	− .167	0.094	102	− .254	0.01	102
Social support—supervisors	− .246	0.013	102	− .282	0.004	102
Social support—co-workers	− .037	0.713	102	− .147	0.14	102
**Well-being**	− .583	0.0001	101	− .323	0.001	101
Well-being—physical	− .367	0.0001	101	− .158	0.115	101
Well-being—psychological	− .665	0.0001	101	− .424	0.0001	101

Correlations between the Exhaustion or Disengagement and the FVTS subscales are presented in Table 3. All of the detected correlations fall in the Weak range. There are correlations between Exhaustion and Fear of the End of Life (r = 0.346, * p* = 0.000), Fear of Life after Death (r = 0.324, *p* = 0.001); Fear of the Process of Dying (r = 0.239, *p* = 0.016), and the GI (r = 0.260, *p* = 0.009).

**Table 3:** Pearson correlations of OLBI scales and FVTS subscales (r—correlation coefficient;*p*—*p*-value; N—sample size)

	Exhaustion			Disengagement		
	r	*p*	N	r	*p*	N
**General index (GI)**	.260	.009	100	.035	.728	100
Fear of meeting with death	.066	.512	100	−.031	.759	100
Fear of mortality	.066	.511	100	−.097	.336	100
Fear of the end of life	.346	.000	100	.068	.504	100
Fear of physical destruction	.012	.904	100	.004	.968	100
Fear of life after death	.324	.001	101	.077	.445	101
Fear of the process of dying	.239	.016	100	.114	.259	100

Tau-b Kendall correlations of OLBI scales and FVTS subscales, and participants’ age and years of working as a transplant coordinator are presented in Table 4. There are weak positive correlations between the age and the GI (r = 0.143, *p* = 0.039), the age and Fear of Physical Destruction (r = 0.196, *p* = 0.006), and years as a transplant coordinator and Exhaustion (r = 0.190, *p* = 0.008).

**Table 4:** Tau-b Kendall correlations of OLBI scales and FVTS subscales, and participants’ age and years of work as a transplant coordinator (r—correlation coefficient; *p*—*p*-value; N—sample size)

	Age			Yrs as transplant coordinator		
	r	*p*	N	r	*p*	N
**Exhaustion**	−.043	.545	103	.190	.008	102
**Disengagement**	−.019	.781	103	.060	.405	102
**General index (GI)**	.143	.039	101	.100	.158	100
Fear of meeting with death	.066	.358	101	.063	.386	100
Fear of mortality	.106	.135	101	.007	.923	100
Fear of the end of life	.099	.164	101	.106	.144	100
Fear of physical destruction	.196	.006	101	.035	.632	100
Fear of life after death	.082	.248	102	.050	.488	101
Fear of the process of dying	.048	.505	101	.131	.074	100

Comparisons of OLBI scale and FVTS subscale means between male and female participants using Student’s t test are presented in Table 5. There are significant differences between male and female means in Disengagement (male M = 18.76, female M = 16.11, t =  − 3.35, *p* = 0.001), GI (male M = 40.42, female M = 47.22, t = 2.12, *p* = 0.036), and Fear of Meeting with Death (male M = 3.33, female M = 5.41, t = 3.19, *p* = 0.002).

**Table 5:** Comparison of OLBI scales and FVTS subscales means between male and female participants (Student’s t test)

Sex	N	M	SD	t	*p*
**Exhaustion**					
♀	70	18.04	3.45	− 1.21	.231
♂	34	18.88	3.06		
**Disengagement**					
♀	70	16.11	3.66	− 3.35	.001
♂	34	18.76	4.03		
**General index (GI)**					
♀	69	47.22	14.86	2.12	.036
♂	33	40.42	15.65		
**Fear of meeting with death**					
♀	69	5.41	3.22	3.19	.002
♂	33	3.33	2.71		
**Fear of mortality**					
♀	69	10.55	3.74	1.89	.062
♂	33	8.94	4.57		
**Fear of the end of life**					
♀	69	7.17	3.82	.22	.825
♂	33	7.00	3.47		
**Fear of physical destruction**					
♀	69	5.25	3.83	.71	.481
♂	33	4.70	3.31		
**Fear of life after death**					
♀	70	6.27	3.94	1.43	.157
♂	33	5.06	4.18		
**Fear of the process of dying**					
♀	69	12.51	3.23	1.49	.140
♂	33	11.39	4.10		

Comparisons of OLBI scale and FVTS subscale means between participants who are single and in relationship using Student’s t test are presented in Table 6. There are significant differences between participants who are in relationship and are single in Exhaustion (relationship M = 18.58, single M = 16.67, t = 2.08, *p* = 0.040), GI (relationship M = 46.38, single M = 37.13, t = 2.19, *p* = 0.031), and Fear of Mortality means (relationship M = 10.49, single M = 7.33, t = 2.87, *p* = 0.005).

**Table 6:** Comparison of OLBI scales and FVTS subscales means between participants who are single and in relationship (Student’s t test)

Status	N	M	SD	t	*p*
**Exhaustion**					
In relationship	88	18.58	3.34	2.08	.040
Single	15	16.67	3.02		
**Disengagement**					
In relationship	88	17.17	4.00	.99	.322
Single	15	16.07	3.02		
**General index (GI)**					
In relationship	87	46.38	15.48	2.19	.031
Single	15	37.13	12.48		
**Fear of meeting with death**					
In relationship	87	4.84	3.21	.79	.434
Single	15	4.13	3.23		
**Fear of mortality**					
In relationship	87	10.49	4.03	2.87	.005
Single	15	7.33	3.29		
**Fear of the end of life**					
In relationship	87	7.38	3.76	1.74	.085
Single	15	5.60	2.97		
**Fear of physical destruction**					
In relationship	87	5.20	3.63	.84	.403
Single	15	4.33	3.92		
**Fear of life after death**					
In relationship	88	6.05	4.17	1.21	.237
Single	15	4.93	3.10		
**Fear of the process of dying**					
In relationship	87	12.38	3.62	1.60	.113
Single	15	10.80	2.93		

## Discussion

In this study, we investigated the experience of professional burnout in a large group of Polish transplant coordinators involved in organ, tissue, and cell deceased donations. Because of the specific work conditions related to deceased donations, we also wanted to examine the relationship between burnout and fear of death and dying, and investigate the differences between men and women, and single and partnered study participants.

The results of the PWC were, in general, similar to those obtained by us previously in a group of employees of cell and tissue banks (Kamiński and Rozenek 2018). In both studies, the results were suggestive of average level of job stress, and the only difference was that in the current study the cognitive job control score was lower than in the cohort studied by us previously. This suggests that in Poland employees of cell and tissue banks present with higher control than transplant coordinators in the area of their specific job duties, access to job-related information, and rules of evaluating their job performance. Our current results also suggest that the studied cohort of donor coordinators obtained results, in terms of job control, social support, and well-being, similar to those of almost 800 Polish nurses studied by Kowalczuk and Krajewska-Kułak (2017). Unfortunately, the authors of that study provided only means for raw scores, and not stens, which makes detailed comparison impossible.

Interestingly, the lowest scores obtained by our sample were those for social support in general and from co-workers. This finding is similar to the results of a survey of Polish transplant coordinators (POLTRANSPLANT 2017) in which coordinators from hospitals with small numbers of reported potential donors identified lack of support from co-workers and supervisors as the most important factor interfering with organ donations.

The outcomes of the PWC, suggestive of average levels of job stress, were consistent with the OLBI results which demonstrated that, in the studied cohort of transplant coordinators, the two components of professional burnout—disengagement and exhaustion—have reached an average level. Even though these results are contrary to the common belief that members of medical professions are especially prone to experiencing burnout, they are corroborated by the published data. The levels of professional burnout obtained for our sample were not only similar to those obtained for employees of cell and tissue banks (Kamiński and Rozenek 2018), but also to those reported by Kim (2013) for liver and kidney transplant coordinators from a large multiorgan transplant center in the Southeast region of the USA. In addition, our results seemed to be lower for exhaustion than those reported for physicians and nurses, and somewhat similar for disengagement to the results obtained by nurses (Innstrand et al. 2011). However, the average exhaustion scores obtained in our study seem to be higher than the low emotional exhaustion results for transplant coordinators reported by Turkish researchers (Harmanci Seren et al. 2013). These differences may probably be explained by the fact that those researchers used a different tool for measuring burnout than we did. In general, detected by us burnout levels seem to overlap with burnout intensity reported in the literature for medical professions. It is worth noting that, according to Maslach et al. (2001), if our sample had included higher number of younger individuals, the overall levels of detected burnout components might have been higher (the mean age for our cohort was 45.32 years). This results from the fact that “those who burn out early in their careers are likely to quit their jobs, leaving behind the survivors who consequently exhibit lower levels of burnout” (Maslach et al. 2001). At the same time, however, in our sample, the exhaustion component of burnout was positively correlated with years of working as a transplant coordinator but not with participants’ age. This may suggest that, in the case of a job as psychologically-demanding as transplant coordinator, the relationship of professional burnout and age may be more complicated than in the case of other, less-stressful professions.

In this study, we detected multiple negative correlations between the two components of burnout measured by the OLBI (exhaustion and disengagement) and different aspects of job control, social support, and well-being as measured by the PWC. The correlations between both aspects of burnout and social support from supervisors reflect the findings reported by us earlier for employees of cell and tissue banks (Kamiński and Rozenek 2018). Similar correlations between social support and burnout have also been reported for hospital-based nurses (Constable and Russel 1986), hospice and critical care nurses (Mallett et al. 1991), Spanish workers (Blanch and Aluja 2012), and university personnel (Charoensukmongkol et al. 2016). For this reason, negative correlations between social support and burnout seem to be a generalized phenomenon which is not specific to medical professions only. However, in our country, lack of social support may be the most important factor impairing the effectiveness of transplantation network (Kamiński and Rozenek 2018; POLTRANSPLANT 2017), and it can be suspected that it exerts its negative influence via increasing burnout in transplantation-involved personnel. Detected by us correlations between exhaustion / disengagement and job control also seem to be universal as other studies found correlations between burnout and different aspects of workplace control in transplantation coordinators (Gruener 2006), physicians (Freeborn 2001), and nurses (Schmitz et al. 2000). Moreover, our results, linking burnout and different aspects of well-being (both psychological and physical), support earlier findings, which detected correlations of professional burnout and psychological health (Ahola et al. 2014; Freeborn 2001; Pompili et al. 2010) and physical health (Cordes and Dougherty 1993; Kim et al. 2011; Leiter 2005; Schaufeli et al. 2009; Toker et al. 2005).

In our sample, men were significantly more disengaged than women. Unfortunately, this observation cannot be put into context and requires further studies as research has produced inconsistent results on sex differences in burnout (Maslach and Jackson 1981; Greenglass and Burke 1988; Adekola 2010; Innstrand et al. 2011; Pu et al. 2017; LaFaver et al. 2018), and it has even been suggested that there may be country-specific differences in burnout between males and females (Purvanova and Muros 2010). Our study participants who were in relationship presented with significantly higher levels of exhaustion than those who were single. As mentioned earlier, exhaustion is predicted by excessive job demands (Demerouti et al. 2001). Yet, in this case, it can be suspected that, because of the determinants specific to Polish culture, majority of those partnered study subjects were married with children, and the demands of supporting the family—on top of job-specific demands—might have contributed to higher exhaustion rates in this population. Similarly, the same cultural factors might have contributed to the higher General Index and Fear of Mortality scores on FVTS observed in partnered study participants. It is possible that higher death-related anxiety in this sample was related to worries stemming from the sense of duty to support their children and families; this is especially clear in the case of the Fear of Mortality subscale which relates to the consequences of non-existence to oneself and related others.

In our study, we detected some positive correlations between different components of fear of death and dying (as measured by the FVTS) and the exhaustion component of professional burnout (as assessed by the OLBI). These findings are similar to the results published by other authors who studied populations of medical professionals. Sliter et al. (2014) found that nurses who had higher levels of death anxiety presented with higher levels of burnout. A relationship between death anxiety and professional burnout has also been reported for health care workers by Melo and Oliver (2011), and for hospice and critical care nurses by Mallett et al. (1991). Because death anxiety tends to remain stable (Linn et al 1982; Kaye and Loscalzo 1998) and, as shown by Rasmussen et al. (1998), it is difficult to achieve change in the levels of death anxiety or death depression, Slitter and collaborators (2014) proposed that “DA [death anxiety] could be assessed by career counselors, who may be able to rule out certain occupations for people who are high in DA” and “career counseling for nurses and doctors could focus on choices between different professional specializations where death is more (e.g., oncology) or less (e.g., rehabilitation) likely to be frequently encountered.” Our findings, linking fear of death and dying with some aspects of professional burnout in transplant coordinators, suggest that similar pre-employment screening could be beneficial also for transplant coordinators candidates as this job will expose them to chronic mortality cues, i.e., chronic external stimuli that serve as a reminder of death (Grant and Wade-Benzoni 2009). This seems to be especially important in the context of a finding that people with high levels of death anxiety were more sensitive to the negative effects associated with mortality cues in terms of burnout (Slitter et al. 2014). It has been proposed that when mortality clues were present, managers should support employee’s death reflection rather than try to sweep the event under the rug (Grant and Wade-Benzoni 2009), as this might evoke death anxiety. The proposal seems to correlate with our previous finding that job-related support from supervisors seemed to be the most important factor which might influence the efficacy of transplantation network in Poland (Kamiński and Rozenek 2018).

In addition, in our current study, some components of the fear of death and dying were positively correlated with participants’ age. This finding, however, cannot be put into a definite context as mixed results have been reported regarding the effects of participants’ age on their death-related anxiety (Bengtson et al. 1977; Kalish and Reynolds 1976; Cicirelli 2002; Suhail and Akram 2002; Tsai et al. 2005). Moreover, we found that levels of some aspects of fear of death and dying were higher in female than in male participants of our study. This finding is consistent with other reports which have found higher levels of death anxiety in females (Hickson et al. 1988; Abdel-Khalek and Tomas-Sabado 2005; Pierce 2007; Ellis et al. 2013).

In general, our results support previously-published reports on professional burnout and its correlates in medical and non-medical populations. It seems to us that, in the case of the population of donor coordinators, the most interesting direction of future studies would be investigating in detail the interactions between death-related anxiety and burnout. We believe that, because of the specific work conditions for transplant coordinators, better understanding of the impact of daily contact with death-related cues could be helpful in preventing burnout in this population.
